# The impact of osteoporosis on arthroscopic rotator cuff repair and postoperative tendon-to-bone healing

**DOI:** 10.3389/fsurg.2025.1683843

**Published:** 2025-10-24

**Authors:** Junlong He, Tao Chen, Chenxi Wu, Kaijia Zhang, Peijie You, Qilong Lai, Hong Jiang, Guanhong Liu

**Affiliations:** Orthopedic Surgery, Suzhou TCM Hospital Affiliated to Nanjing University of Chinese Medicine, Suzhou, Jiangsu, China

**Keywords:** osteoporosis, rotator cuff injury, shoulder arthroscopy, tendon-to-bone healing, retear

## Abstract

This review explores the impact of osteoporosis on arthroscopic rotator cuff repair and subsequent tendon healing, focusing on challenges such as anchor fixation failure, intraoperative fractures, and limited surgical visibility. It examines how osteoporosis disrupts the tendon healing microenvironment post-surgery through mechanisms involving bone metabolism, growth factors, the immune system, sex hormones, oxidative stress, and adipose infiltration. Effective surgical planning is crucial to mitigate the adverse effects of osteoporosis on rotator cuff repair. This review offers recommendations for optimizing surgical strategies, including anchor selection, placement, and fixation techniques. In addition, it highlights the potential of anti-osteoporotic drugs and biological therapies to improve tendon-to-bone union and enhance clinical outcomes. For cases of inevitable repair failure, remedial strategies are proposed to inform clinical practice. A systematic literature search was conducted in the PubMed, Web of Science, and CNKI databases (2000–2025) using the following keywords: “osteoporosis,” “rotator cuff injury,” “shoulder arthroscopy,” “tendon-to-bone healing,” and “retear.” The inclusion criteria were as follows: (1) human or animal studies; (2) MRI-confirmed rotator cuff tears; and (3) full-text articles in English or Chinese. The exclusion criteria included case reports (*n* < 10).

## 1 Introduction

Rotator cuff injury is a prevalent shoulder pathology characterized by pain and functional impairment, particularly in middle-aged and elderly populations (1). Due to its limited capacity for self-healing, rotator cuff injuries frequently require surgical intervention. Arthroscopic shoulder surgery is widely used to treat such injuries, although the risk of postoperative retears remains significant (2). Osteoporosis, which is characterized by reduced bone mass and deteriorated bone microarchitecture, represents a metabolic imbalance in bone tissue, resulting in increased fragility and fracture risk. It is both a risk factor for the initial occurrence of rotator cuff injuries and a factor that adversely affects postoperative tendon-to-bone healing, thereby increasing the likelihood of retears (3, 4). Clinically, patients with rotator cuff injuries and osteoporosis face greater surgical challenges, an elevated risk of impaired tendon-to-bone healing, and subsequent retears that may necessitate revision surgery, significantly increasing the economic burden (5). Recent research has focused on improving surgical outcomes, optimizing the tendon-to-bone healing microenvironment, and reducing the postoperative retear rate. With advancements in medical technology, clinicians have explored new strategies in surgical techniques and bioengineering, offering promising pathways for enhanced prognosis. This review aims to analyze the impact of osteoporosis on arthroscopic rotator cuff repair and postoperative tendon-to-bone healing, while also discussing current clinical strategies for optimizing outcomes.

## 2 Relationship between osteoporosis and rotator cuff injury

Osteoporosis increases the risk of rotator cuff injury. In a matched cohort study by Hong et al. (4), 17,067 osteoporotic patients and 100,501 non-osteoporotic controls were followed for 7 years. The results revealed 166 and 89 cases of rotator cuff tears in the osteoporotic and non-osteoporotic groups, respectively, indicating a 1.79-fold higher risk of rotator cuff tear among osteoporotic patients compared with their non-osteoporotic counterparts. Age-related decreases in bone mineral density (BMD), particularly in the proximal humerus and greater tuberosity, complicate the surgical repair of rotator cuff injuries. Reduced BMD at these critical sites alters the tendon-to-bone healing microenvironment, increasing the risk of postoperative retear. In addition, decreased local mechanical stimulation from the rotator cuff injury may contribute to secondary osteoporosis (5, 6). Chen et al. (7) categorized 74 rotator cuff injury patients into three groups based on preoperative bone density (normal bone mass, osteopenia, and osteoporosis). After 12 months of postoperative follow-up, significantly higher retear rates were observed in the osteopenic and osteoporotic groups compared with the normal bone density group. Lee et al. (8) performed dual-energy x-ray absorptiometry (DEXA) on 87 patients with unilateral rotator cuff injuries and analyzed the BMD of various regions of interest (ROIs), including the humeral head, lesser tuberosity, medial greater tuberosity, middle greater tuberosity, lateral greater tuberosity, and the overall proximal humerus. Their results demonstrated significantly lower BMD in all ROIs on the injured side compared with the contralateral healthy side (all *p* < 0.05).

Thus, thorough osteoporosis screening is essential for middle-aged and elderly patients with rotator cuff injuries, and timely intervention should be implemented for those diagnosed with osteoporosis.

## 3 Influence of osteoporosis on rotator cuff repair surgery

### 3.1 Increased risk of anchor fixation failure

Patients with osteoporosis face greater challenges in achieving stable anchor fixation compared with those without osteoporosis. Localized osteoporosis frequently leads to early postoperative anchor loosening or dislodgement, resulting in long-term instability of the bone bed. This instability compromises the healing microenvironment and increases the risk of rotator cuff retears (6). Tingart et al. (9) conducted a cadaveric study to examine the relationship between proximal humeral BMD and anchor pull-out strength. They found a significant positive correlation, highlighting the need for thorough preoperative osteoporosis evaluation, optimization of surgical techniques, careful implant selection, and real-time intraoperative monitoring of anchor stability to mitigate the risk of postoperative retears.

### 3.2 Increased risk of intraoperative fracture

The risk of intraoperative fractures in osteoporotic patients primarily results from reduced bone strength combined with concentrated mechanical stress during surgery. Shoulder arthroscopy is typically performed with the patient in a lateral decubitus position, maintaining prolonged arm abduction to maximize the space between the humerus and glenoid (10). However, in osteoporotic patients, excessive abduction traction may predispose the proximal humerus to fractures. In addition, localized compression during cannula insertion can cause fractures at the entry site. Careful management of the footprint bone bed is also critical, as excessive abrasion in this area can disrupt the subchondral bone plate, potentially leading to localized humeral head collapse (5). Furthermore, careful drilling of anchor pilot holes and precise control of suture tension are essential, as excessive drilling speed and tension may contribute to further localized fractures.

### 3.3 Limited surgical visualization

Patients with osteoporosis often experience reduced bone density, which leads to weakened capsular attachment sites or humeral head joint surface collapse, resulting in joint misalignment. The passive stretching of the capsule required to maintain joint stability can cause capsular laxity, negatively affecting surgical visualization. Restricted visibility prolongs the operative time, increasing the risk of infection. In addition, diminished visibility compromises surgical precision, raising the risk of incorrect anchor placement or inadvertent damage to neurovascular structures and tendons. Higher irrigation pressures, necessary to maintain joint space expansion, also increase the risk of fluid extravasation, potentially causing localized limb swelling and adverse effects on peripheral vasculature (11). Therefore, continuous intraoperative monitoring of skin tension and limb swelling is essential to avoid excessive irrigation pressures and related complications.

## 4 Influence of osteoporosis on postoperative tendon-to-bone healing

**Figure 1:**
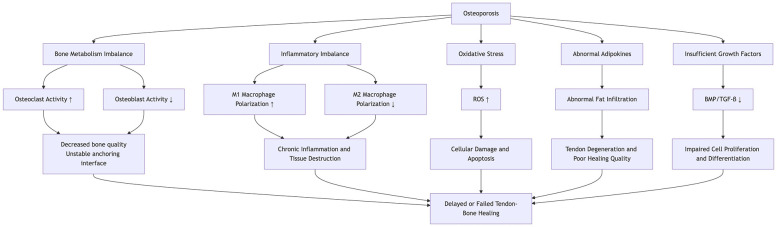
This diagram illustrates the mechanisms by which osteoporosis affects the healing process of the tendon-bone, involving bone metabolism, inflammation, oxidative stress, adipokines, and growth factors.

The normal tendon-to-bone junction (enthesis) consists of a highly specialized tissue architecture, including bone, calcified fibrocartilage, non-calcified fibrocartilage, and tendon, which effectively distributes mechanical stress between the tendon and bone, thereby enhancing biomechanical properties (12). Surgical intervention is the primary treatment for injuries at the tendon-to-bone interface. However, clinical studies indicate that surgically repaired tendon-to-bone junctions typically heal as disorganized scar tissue, rather than restoring the original four-layer structure. The irregular extracellular matrix and reduced elasticity of scar tissue significantly impair biomechanical function (13). In patients with osteoporosis, the altered healing microenvironment often results in poor tendon-to-bone integration, significantly increasing the risk of postoperative retears (3). Key mechanisms by which osteoporosis affects tendon-to-bone healing (Figure 1) include the following.

### 4.1 Imbalanced bone metabolism

Normal bone metabolism results from the precise coordination between osteoclasts and osteoblasts, maintaining skeletal integrity through the continuous resorption of old bone and formation of new bone. In osteoporosis, the dynamic balance between bone resorption and formation is disrupted; osteoclast activity is enhanced, accelerating bone resorption, while osteoblast function, particularly the differentiation and activity of mesenchymal stem cells (MSCs), is suppressed. This imbalance directly impacts the tendon-to-bone interface. Xu et al. (14), using an osteoporotic rat model, investigated the effect of osteoporosis on tendon-to-bone healing after rotator cuff repair. They found that early postoperative healing at the tendon-to-bone interface was impaired due to heightened osteoclast activity. MSCs exert multiple regulatory functions through exosome secretion. In a rat model of rotator cuff reconstruction, Huang et al. (15) demonstrated that bone marrow MSC-derived exosomes (BMSC-Exos) increased failure load and stiffness at the repair site, induced angiogenesis at the tendon-to-bone interface, and promoted healing. However, in osteoporosis, MSC function is often abnormal, with a shift toward enhanced adipogenic differentiation and reduced osteogenic and chondrogenic differentiation (16), thus hindering fibrocartilage regeneration at the interface and impairing effective tendon-to-bone healing.

### 4.2 Growth factor function is inhibited

Growth factors are cytokines or proteins that play pivotal roles in cell differentiation throughout the healing process. In tendon-to-bone healing, key growth factors such as bone morphogenetic proteins (BMPs), transforming growth factor (TGF), vascular endothelial growth factor (VEGF), and fibroblast growth factor (FGF) regulate cell proliferation, differentiation, and extracellular matrix synthesis, promoting fibrocartilage regeneration and angiogenesis, which are essential for effective tendon-to-bone integration (17). In the pathological microenvironment of osteoporosis, the normal regulatory functions of growth factors may be impaired, or their associated signaling pathways may be disrupted (18, 19), potentially leading to delayed healing at the tendon-to-bone interface or reduced structural strength. Clinically, growth factors can be used as adjuncts to enhance repair; however, their therapeutic outcomes remain inconsistent, influenced by factors such as delivery method, dosage, and timing of application. Achieving precise, controlled local release of growth factors to optimize their effects in the microenvironment remains a key challenge for future research.

### 4.3 Decreased sex hormone levels

Previous studies have shown a strong association between sex hormones and tendon-to-bone healing. Tashjian et al. (20) investigated the effects of estrogen and testosterone supplementation in male mice following rotator cuff repair. Histological analysis at 8 weeks post-surgery revealed that supplementation with either estrogen or testosterone significantly improved tendon healing quality. Similarly, Tanaka et al. (21), using an ovariectomized rat model, examined the impact of estrogen deficiency on tendon-to-bone healing after rotator cuff repair. Their results indicated that insufficient estrogen impaired fibrocartilage-like tissue formation at the tendon-to-bone interface, thereby hindering the healing process.

Therefore, in postmenopausal women or elderly male patients, it may be beneficial to assess sex hormone levels and, when appropriate, consider hormone replacement therapy or other endocrine interventions to improve the tendon-to-bone healing microenvironment.

### 4.4 Persistent low-grade immune activation

Tendon-to-bone healing progresses through three overlapping phases: inflammation, repair, and remodeling. The inflammatory phase is crucial, with macrophages playing an active role throughout the process. During this phase, macrophages polarize into the M1 phenotype, secreting pro-inflammatory cytokines such as interleukin-1β (IL-1β) and tumor necrosis factor-α (TNF-α) to mediate inflammation, clear necrotic tissue, and promote fibroblast proliferation. In the repair and remodeling phases, macrophages shift toward the M2 phenotype, producing anti-inflammatory cytokines such as interleukin-10 (IL-10) and TGF-β to suppress inflammation and facilitate tissue repair (22, 23). Osteoporosis is often associated with persistent low-grade immune activation, characterized by a higher M1/M2 macrophage ratio and a shift toward M1 polarization. This leads to the accumulation of pro-inflammatory cytokines, intensifying local inflammation, which hinders tendon-to-bone healing (24–26). In addition, excessive early postoperative inflammation may further impair the healing microenvironment. To mitigate the negative effects of excessive inflammation, non-steroidal anti-inflammatory drugs (NSAIDs), cryotherapy, laser therapy, and other physical modalities may help reduce local inflammation and improve postoperative outcomes.

### 4.5 Oxidative stress

Oxidative stress occurs when there is an imbalance between the production of reactive oxygen species (ROS) and the body's antioxidant defense systems, leading to excessive ROS accumulation, cellular damage, apoptosis, and impaired cellular function (27). Clinical studies have shown that patients with osteoporosis exhibit significantly lower total serum antioxidant capacity and higher serum peroxide levels (28). In another study, postmenopausal women were classified into normal bone mass, osteopenia, and osteoporosis groups based on BMD. Serum measurements revealed that catalase, superoxide dismutase 2 (SOD2), and peroxiredoxin 2 (PRX2) levels were significantly reduced in the abnormal bone mass groups compared with the normal group (29). This pathological imbalance between oxidative and antioxidative systems negatively impacts tendon-to-bone healing. Itoigawa et al. (2) found that oxidative stress and superoxide dismutase (SOD) levels were significantly higher in patients with postoperative retears compared with those with successful tendon healing after arthroscopic rotator cuff repair. Uehara et al. (30), using a rat rotator cuff repair model, demonstrated that antioxidant treatments, such as N-acetylcysteine (NAC) and vitamin C (VC), reduced oxidative stress at the repair site and accelerated the healing process.

### 4.6 Fatty infiltration

Studies have shown that osteoporosis is often accompanied by fatty infiltration (31–33). Fatty infiltration of shoulder musculature can impair fibrocartilage formation at the tendon-to-bone interface, leading to poor healing outcomes (3, 34). Yang et al. (35) reported that fatty infiltration of the rotator cuff muscles, particularly the infraspinatus, significantly increased the risk of postoperative retears and was associated with lower shoulder function scores. Li et al. (36), using a rat rotator cuff injury model, demonstrated that overexpression or knock-out of the ubiquitin ligase NEDD4 could regulate adipocyte differentiation and lipid metabolism, thereby reducing fatty infiltration and promoting tendon-to-bone healing. Therefore, prolonged immobilization of the shoulder joint after a rotator cuff injury should be avoided to prevent disuse osteoporosis and fatty infiltration. For patients already experiencing fatty infiltration, pharmacological interventions targeting lipid metabolism may be considered.

### 4.7 Vitamin D deficiency

Vitamin D, particularly its active form 1,25-dihydroxyvitamin D₃, enhances the intestinal absorption of calcium and phosphorus, providing essential substrates for bone mineralization, and works synergistically with parathyroid hormone (PTH) to regulate bone metabolism and maintain serum calcium homeostasis. Vitamin D deficiency is detrimental to tendon-to-bone healing. Chen et al. (37) included 89 patients with full-thickness rotator cuff tears undergoing arthroscopic repair, categorizing them into control and deficiency groups based on serum vitamin D levels. The results showed that the deficiency group had a significantly higher retear rate compared with the control group and was more prone to early postoperative pain. As osteoporosis is frequently accompanied by vitamin D deficiency, combined supplementation with calcium and vitamin D during treatment may help improve bone quality and promote tendon-to-bone healing.

## 5 Strategies to improve prognosis

**Figure 2:**
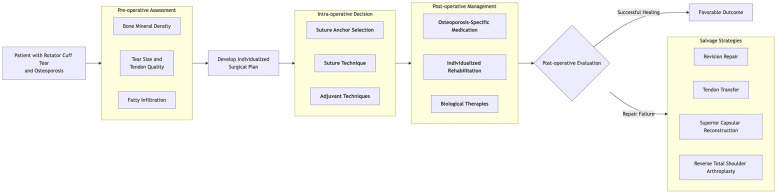
This diagram illustrates the clinical decision pathway for patients with rotator cuff injuries complicated by osteoporosis.

To mitigate the impact of osteoporosis on rotator cuff repair and improve outcomes, current approaches focus on two main strategies: first, optimizing surgical techniques to prevent anchor pull-out, and second, enhancing bone quality at fixation sites through osteoporosis medication or biological therapies to promote tendon-to-bone union (Figure 2).

### 5.1 Enhancing stability of internal fixation

#### 5.1.1 Increase the number of anchor points

Increasing the number of fixation points can effectively distribute stress across the bone–anchor interface, reducing stress concentration at the tendon-to-bone junction, lowering the risk of anchor loosening or failure, and improving anchor pull-out strength (5). However, the number of anchors is limited by the tear size and footprint area. Research suggests a minimum distance of 6 mm between anchors to maintain pull-out strength; therefore, anchors should be spaced at least 6 mm apart during placement (38). Clinicians should carefully evaluate tendon tear size, footprint dimensions, and local bone quality, as excessive anchor placement may prolong surgery, increase the risk of infection, and raise the patient's economic burden.

#### 5.1.2 Single-row, double-row, and suture bridge techniques

The suture technique in rotator cuff repair is crucial for reattaching the tendon to the greater tuberosity. Common methods include single-row, double-row, and suture bridge techniques. Gu et al. (39) reported significantly lower retear rates with double-row techniques compared with single-row techniques. However, they also found no significant difference in outcomes between single- and double-row techniques for tears smaller than 3 cm. The suture bridge technique, an enhanced variation of double-row suturing, has demonstrated improved clinical outcomes in previous studies (40).

#### 5.1.3 Appropriate anchor specification and type

The mechanical fixation strength (pull-out strength) of suture anchors depends on factors such as thread pitch, thread count, length, dimensions, and anchor geometry. Various anchor designs, materials, and dimensions have been developed, each exhibiting different biomechanical performances (41). Chae et al. (42) identified that increasing anchor length, thread count, thread height, and the contact area between anchor threads and surrounding bone enhances anchor pull-out strength. Clinically, commonly used anchors include metal anchors, polymer anchors [e.g., polyetheretherketone (PEEK)], bioabsorbable anchors, biocomposite anchors, and all-suture anchors. Yang et al. (43) conducted biomechanical comparisons of different anchor types used in rotator cuff repairs. They found that PEEK anchors exhibited the highest ultimate failure load, while biocomposite anchors had the lowest. All-suture anchors demonstrated the highest stiffness, while PEEK anchors had the lowest stiffness. Regarding displacement, metal anchors exhibited minimal displacement, followed by all-suture anchors. Importantly, they noted that BMD significantly influences anchor performance. All-suture anchors performed superiorly in osteoporotic bone models. This is primarily due to their superior stress dispersion capabilities, lower bone volume requirements, and reduced bone intrusion. Unlike traditional anchors, the full-thread anchor evenly distributes tensile forces through high-strength suture material, minimizing stress concentration in fragile bone areas, thereby reducing the risk of anchor loosening or fracture. Simultaneously, its compact size and design result in minimal bone intrusion, reducing the risk of bone damage. Moreover, it does not rely on substantial bone volume for stability. Instead, it capitalizes on the relatively well-preserved mechanical properties of cortical bone in osteoporotic skeletons, ensuring effective fixation even in osteoporotic patients and guaranteeing the stability of the repair. Therefore, in osteoporotic patients, the use of all-suture anchors should be considered if local bone conditions permit.

The angle of anchor implantation is also crucial. Liu et al. (44) reported that, at equivalent bone densities, anchors implanted perpendicularly (at a 90° angle) significantly enhanced biomechanical stability. In addition, perpendicular implantation facilitates suture knotting during surgery and improves postoperative recovery of the supraspinatus muscle.

#### 5.1.4 Bone grafting and cement augmentation

During arthroscopic rotator cuff repair, bone grafting or cement augmentation can effectively address defects caused by osteoporotic bone resorption or subchondral cystic lesions. Levy et al. (45) compressed cancellous allograft bone into proximal humeral cystic defects, creating a robust bone bed for anchor fixation and enhancing fixation strength. Fang et al. (46) demonstrated that autologous osteochondral tissue and periosteal grafts significantly promoted fibrocartilage regeneration at the tendon-to-bone interface. However, arthroscopic bone grafting remains technically challenging and is unsuitable for osteoporosis without significant bone defects. Therefore, clinical preference often favors injectable bone cement for bone augmentation and improved anchor fixation strength. Aziz et al. (47) demonstrated in cadaveric studies that polymethylmethacrylate (PMMA) cement significantly enhanced anchor pull-out strength. However, the non-absorbable nature of PMMA complicates revision surgeries and poses a risk of intra-articular extravasation during injection. In addition, thermal effects during cement curing may lead to osteonecrosis. Novel bioabsorbable fiber-reinforced bone cements offer similar tensile strength to PMMA without thermal effects and are absorbable, representing promising enhancement materials (48). Further research into the biocompatibility and safety of the degradation products from these new cements is needed.

#### 5.1.5 Patch augmentation techniques

Patch augmentation involves placing graft materials around torn rotator cuff tendons to provide mechanical support, distribute stress, and promote tissue healing. Both synthetic and biological patches are currently used clinically. Wang et al. (49) demonstrated improved biomechanical properties and tendon-to-bone healing using decellularized amniotic membrane (DAM) in a rat supraspinatus tendon tear model. Similarly, another study (50) showed that acellular amniotic membrane (AAM) facilitated tendon-to-bone healing more effectively when interposed between the tendon and bone, rather than when merely overlaid. Synthetic patches may induce chronic inflammation or infections due to poor tissue compatibility, potentially leading to repair failure (51). Biological patches carry the risk of immunological rejection, and mismatched degradation rates may negatively affect repair outcomes. In addition, decellularized tendon grafts face unresolved issues, such as inferior biomechanical properties compared with normal tendons post-decellularization (52). Therefore, further research is needed to enhance the properties of these biomaterials for improved tendon repair.

#### 5.1.6 Optimization of footprint management

Proper management of the tendon footprint is crucial for ensuring anchor stability and promoting favorable tendon-to-bone healing. Hyatt et al. (53) studied human humeral specimens to examine the biomechanical impact of cortical bone decortication on anchor fixation. The results showed significantly reduced anchor pull-out strength in decorticated specimens (62.84 ± 38.04 N/mm) compared with non-decorticated specimens (244.04 ± 89.06 N/mm; *P* < 0.0001). Therefore, excessive cortical bone decortication at the footprint should be avoided, particularly in osteoporotic patients. In addition, Sun et al. (54) demonstrated in a rabbit rotator cuff tear model that preserving remnant tendon tissues significantly enhanced biomechanical and histological outcomes, improving overall rotator cuff healing.

### 5.2 Mitigating the effects of osteoporosis on tendon-to-bone healing

#### 5.2.1 Application of anti-osteoporosis medications

Postoperative administration of anti-osteoporosis medications can enhance tendon-to-bone healing following rotator cuff repair. Current medications for osteoporosis include foundational treatments (e.g., calcium and vitamin D), antiresorptive agents (e.g., bisphosphonates, RANKL inhibitors), anabolic agents (e.g., PTH analogues), and alternative treatments (e.g., strontium salts, traditional Chinese medicines, and their extracts) (55). Zhao et al. (56) demonstrated in a retrospective study that intravenous zoledronic acid significantly reduced retear rates after rotator cuff repair in elderly osteoporotic patients. Xu et al. (57) explored the effects of abaloparatide (ABL) and denosumab (Dmab) on tendon-to-bone healing using an osteoporotic rat model with chronic rotator cuff tears, finding both drugs beneficial, with ABL's anabolic effects yielding superior outcomes compared with Dmab's antiresorptive effects. However, discontinuing anti-osteoporosis medication may lead to deterioration in bone quality. This “bone deterioration” refers not to an absolute decline in bone quality, but to the gradual weakening or even disappearance of the benefits provided by the medication—specifically, the increase in bone density and reduction in fracture risk. Therefore, determining an appropriate treatment duration and implementing regular monitoring is crucial. Treatment cycles are not indefinite but are planned based on pharmacokinetics and the patient's fracture risk. For example, bisphosphonates enter an evaluation phase after an initial 3–5 years of treatment. During this period, regular bone density monitoring and biomarker testing can reflect drug efficacy and bone metabolic status, helping clinicians adjust treatment regimens. Reports have also indicated that osteoporosis medications can cause adverse reactions such as osteonecrosis of the jaw and hypocalcemia. Thus, individualized treatment, considering patient-specific bone quality and overall health status, is essential.

#### 5.2.2 Biological therapies

Biological therapies include cellular treatments [platelet-rich plasma (PRP), stem cell therapy], growth factors, scaffolds, and gene therapy. PRP contains multiple growth factors that can enhance tendon-to-bone healing after rotator cuff repair. Peng et al. (58) reported reduced retear rates and improved clinical outcomes with PRP during arthroscopic rotator cuff repairs. However, further exploration is needed regarding optimal PRP concentration, bioactive component mechanisms, and the timing of application. Scaffold technologies, which serve as carriers for growth factors and stem cells, can activate repair potential at the injury site, providing temporary mechanical support and promoting organized regeneration through biomimetic structures (17). Advances in 3D printing have enhanced scaffold fabrication, allowing the creation of bioactive scaffolds that replicate natural tendon structure, maintain mechanical strength, and improve cellular communication and tendon-to-bone integration (59). Ni et al. (60) developed a 3D-printed polycaprolactone (PCL) scaffold loaded with basic FGF and bone marrow mesenchymal stem cells (BMSCs), significantly improving biomechanical strength, histological scores, and local bone density 2 weeks post-surgery in a rat rotator cuff tear model. Nevertheless, precise control of scaffold degradation rates to match tissue regeneration remains challenging, and immunological reactions requiring prolonged immunosuppression are potential drawbacks. Gene therapy involves targeting osteogenic genes (e.g., Runx2 and Osterix) to the tendon-to-bone interface via adenovirus or liposomes. Xie et al. (61) used an adenoviral vector carrying the Runx2 gene to transfect human amniotic mesenchymal stem cells (hAMSCs), directing differentiation toward ligament fibroblasts and enhancing tendon-to-bone healing in a rabbit anterior cruciate ligament (ACL) reconstruction model. However, the risk of viral integration into host genomes, leading to uncontrolled gene expression, remains a concern.

### 5.3 Appropriate postoperative rehabilitation

Postoperative rehabilitation is critical following rotator cuff repair. Yoo et al. (62) randomly assigned 75 patients to early or delayed rehabilitation protocols and found no significant differences between the groups in postoperative range of motion, functional outcomes, muscle strength recovery, or tendon healing during short- and midterm follow-ups. Therefore, rehabilitation strategies should balance the risk of fixation failure associated with early rehabilitation against the risk of joint stiffness from delayed rehabilitation. Patient-specific factors, such as bone quality, tear size, and fixation technique, are crucial. In addition, combining extracorporeal shockwave therapy with rehabilitation exercises has shown superior outcomes in reducing early postoperative shoulder pain and accelerating tendon healing at anchor sites compared with rehabilitation alone (63).

## 6 Salvage strategies following failed rotator cuff repair

When anchor fixation failure or retear occurs postoperatively, timely salvage strategies should be implemented. For patients with good tendon quality, if significant anchor loosening is observed, the loose anchors should be removed, and new anchor points should be selected for refixation. For anchors with minimal loosening, they may be retained and supplemented with additional anchors for reinforced fixation (64). Intraoperatively, adjustments to suture techniques, alternative anchor types, or cement augmentation can be considered to reattach the tendon. However, revision surgery carries a higher risk of infection, and scar tissue or adhesions from the initial procedure may complicate surgery.

For poor tendon quality, where re-suturing is not feasible, alternative options include tendon transfer procedures such as latissimus dorsi transfer (LDT) or pectoralis major transfer (PMT), superior capsular reconstruction (SCR), or reverse total shoulder arthroplasty (RTSA). LDT is primarily indicated for irreparable posterosuperior rotator cuff tears, with studies showing that arthroscopy-assisted LDT can restore flexion and abduction comparable to the asymptomatic contralateral shoulder and healthy controls (65). PMT is effective for irreparable subscapularis tears, improving anterior shoulder stability and internal rotation strength. SCR, suitable for massive irreparable tears without severe glenohumeral arthritis, uses autograft or allograft tissue to reconstruct the superior capsule, restoring humeral head stability, reducing superior migration, and improving shoulder function (66). Mihata et al. (67) followed patients for 10 years after SCR using autologous fascia lata grafts, reporting high rates of return to sports and work with sustained clinical and structural improvements.

Subacromial balloon spacer implantation has emerged as a novel approach, involving the insertion of an absorbable balloon in the subacromial space. This device cushions the humeral head from the acromion during deltoid activation and arm abduction, potentially improving biomechanics and reducing pain (68, 69). However, clinical results have been mixed. Verma et al. (70) randomized 184 patients with massive irreparable posterosuperior tears to balloon implantation or partial repair, finding that the balloon could substitute for partial repair, offering superior early functional recovery and pain relief. In contrast, Haque et al. (71) reported no additional benefit of balloon implantation over arthroscopic debridement alone in a randomized controlled trial. Therefore, the clinical effectiveness of subacromial balloon spacers warrants further investigation.

For irreparable rotator cuff tears with concurrent joint degeneration, RTSA is often the preferred option. While it does not restore the rotator cuff, it effectively relieves pain and, through the “ball-and-socket reversal” design, medializes and lowers the center of rotation, enabling the deltoid muscle-particularly its anterior and middle fibers to compensate for lost abduction function. Even in the complete absence of supraspinatus and infraspinatus function, this allows restoration of arm elevation. However, osteoporosis-related risks, such as prosthesis loosening and periprosthetic fractures, remain important considerations.

## 7 Conclusion

Arthroscopic treatment of rotator cuff injuries complicated by osteoporosis presents dual challenges: osteoporosis not only increases the risk of injury but also disrupts the postoperative tendon-to-bone healing microenvironment, leading to anchor loosening, high retear rates, and suboptimal functional recovery. Current strategies, including optimization of anchor fixation techniques, the use of anti-osteoporosis medications, and the application of biological therapies, have improved repair stability and healing quality. In addition, salvage options such as revision repair and tendon transfer provide solutions for failed cases. However, systemic anti-osteoporosis therapies lack precise regulation of the local healing microenvironment, and biomaterials still face challenges related to compatibility and degradation rate matching. Moreover, individualized surgical planning requires more objective guidance from reliable biomarkers.

To address these challenges, future research should focus on several key areas aimed at precisely regulating the local microenvironment and optimizing therapeutic strategies. First, locally targeted drug delivery systems will become a major research direction. For example, developing injectable hydrogels loaded with bisphosphonates or teriparatide will enable precise local drug release, synergistically inhibiting bone resorption and promoting regeneration to improve tendon healing. In addition, novel composite materials hold immense potential, particularly biomimetic mineralized collagen scaffolds and nanobioceramic composites. These materials offer superior osseointegration and mechanical strength, while also modulating local bioactivity to accelerate postoperative healing. Beyond material innovations, precision medicine will play a pivotal role in future research. Integrating imaging techniques with serum biomarkers will enable the development of personalized postoperative prognosis prediction models, guiding the formulation of tailored treatment and rehabilitation plans.
